# Effect of antioxidant supplementation containing L-carnitine on semen parameters: a prospective interventional study

**DOI:** 10.5935/1518-0557.20200043

**Published:** 2021

**Authors:** Leila Nazari, Saghar Salehpour, Sedighe Hosseini, Farzad Allameh, Ferdoos Jahanmardi, Elham Azizi, Robabeh Ghodssi-Ghassemabadi, Teibeh Hashemi

**Affiliations:** 1 Department of Obstetrics and Gynecology, Preventative Gynecology Research Center, Shahid Beheshti University of Medical Sciences, Tehran, Iran; 2 Department of Urology, Preventative Gynecology Research Center, Shahid Beheshti University o f Medical Sciences, Tehran, Iran; 3 Department of Biology and Anatomical Sciences, Student Research Committee, Shahid Beheshti University of Medical Sciences, Tehran, Iran; 4 Department of Biostatistics, Faculty of Medical Sciences, Tarbiat Modares University, Tehran, Iran

**Keywords:** antioxidant supplementation, semen analysis, oxidative stress, reactive oxygen species (ROS)

## Abstract

**Objective:**

One of the remarkable causes of infertility in men is oxidative stress having a reducing effect on their reproductive function. In the present study, we investigated the efficacy of supplementation with antioxidants and L-Carnitine (contained in Androferti) on semen parameters.

**Methods:**

We included 180 infertile male patients diagnosed with idiopathic oligoastenoteratozoospermia (OAT) in this study, and we analyzed the semen sample from 59 patients before and after oral antioxidant treatment, with the commercial name of Androferti (containing 1500 mg of L-Carnitine, 60 mg of vitamin C, 20 mg of coenzyme Q10, 10 mg of vitamin E, 10 mg of zinc, 200 µg of vitamin B9, 50 µg of selenium, 1 µg of vitamin B12). All of the patients received Androferti twice a day for 3 months.

**Results:**

There were significant improvements in the sperm concentration (*p*=0.004) after the antioxidant supplementation. There was also a meaningfully improvement in sperm morphology (*p*=0.01) after treatment. However, sperm motility was not significantly altered after antioxidant treatment (*p*=0.2).

**Conclusions:**

Antioxidants supplementation containing 1500 mg L-carnitine can improve the semen quality in infertile men diagnosed with idiopathic OAT. However, further studies are required to determine the antioxidant effects on reproduction function.

## INTRODUCTION

Infertility is a global problem that affects about 15% of couples intending to conceive ^(Practice Committee of American Society for Reproductive Medicine, 2013)^. The World Health Organization (WHO) defines infertility as the inability of couples to achieve a successful pregnancy after 12 months of regular intercourse using no contraception ^(Practice Committee of American Society for Reproductive Medicine, 2013)^. Male factor infertility is one of the common causes of pregnancy failure in infertile couples. Idiopathic male infertility, which involves the majority of infertile men, affects the semen quality without an identifiable cause. The improvement in semen quality is still a challenge in the treatment of these men ^(Agarwal et al., 2019)^.

Reactive oxygen species (ROS) have been proposed as fundamental factors that may negatively affect semen quality ^(Tremellen, 2008)^. ROS are produced as the end products when a cell uses oxygen, and their increased levels can lead to cellular damage ^(Tremellen, 2008)^. Every human body is naturally equipped with the ability to neutralize the excess ROS, which can be produced either due to endogenous or exogenous factors ^(Smits et al., 2018)^. Any disturbance in the balance between ROS and antioxidants can inevitably lead to oxidative stress. Oxidative stress is defined as the imbalance between the ROS production and the body’s ability to detoxify them and a considerable contributor to the pathophysiology of male infertility ^(Behrman et al., 2001)^. Several exogenous factors (e.g., environmental pollutions, smoking, alcohol consumption, and poor nutrition) and endogenous factors (e.g., obesity, infections, and chronic and autoimmune diseases) are mentioned in the literature as the most common causes of oxidative stress in the reproductive tract ^(Tremellen, 2008)^. Oxidative stress has been reported in most of the infertile men. Although a physiological level of ROS is essential for sperm maturation, capacitation, hyper-activation, and fertilization, oxidative stress can lead to detrimental effects on spermatogenesis and semen quality ^(Rajesh Kumar et al., 2002^; ^Guthrie & Welch, 2012)^. Antioxidants are agents that prevent oxidative damage to cells and tissues via directly scavenging and inactivating excessive ROS and repairing its damages ^(Mirończuk-Chodakowska et al., 2018)^. Some of the well-known enzymatic and non-enzymatic agents which act as antioxidants in the human body are catalase, glutathione reductase, superoxide dismutase, glutathione peroxidase, ascorbic acid, alpha-tocopherol, ferritin, and transferrin ^(Gupta et al., 2007)^.

L-carnitine is one of the natural antioxidants existing in - namely - the seminal plasma of mammals, that inactivate ROS, inhibit lipid peroxidation, and protect the sperm membrane ^(Gülçin, 2006^; ^Ahmed et al., 2017)^. This antioxidant is mainly secreted from mammalian epithelium into the epididymal plasma, and it finally builds up in the sperm cells and protects them from oxidant injury ^(Ahmed *et al*., 2017)^. Moreover, L-carnitine is an essential co-factor for beta-oxidation, which leads to energy production from lipids in the mitochondria ^(Ahmed *et al*., 2017)^. Additionally, L-carnitine is an inexpensive natural antioxidant, and almost free of side effects ^(DiNicolantonio et al., 2019)^. Hence, L-carnitine supplementation has been proposed to be useful for the treatment of infertile men ^(Agarwal et al., 2004^; ^Balercia et al., 2005^; ^Garolla et al., 2005^; ^Cavallini et al., 2004^; ^Lenzi et al., 2004)^. However, the efficacy of L-carnitine treatment in patients diagnosed with idiopathic oligoastenoteratozoospermia (OAT) is still under debate. The present study aimed to assess the influence of an antioxidant-containing supplementation (containing 1500 mg of L-Carnitine, 60 mg of vitamin C, 20 mg of coenzyme Q10, 10 mg of vitamin E, 10 mg of zinc, 200 µg of vitamin B9, 50 µg of selenium, 1 µg of vitamin B12) on the semen parameters of infertile men with idiopathic OAT.

## MATERIALS AND METHODS

### Study population

**Figure 1:**
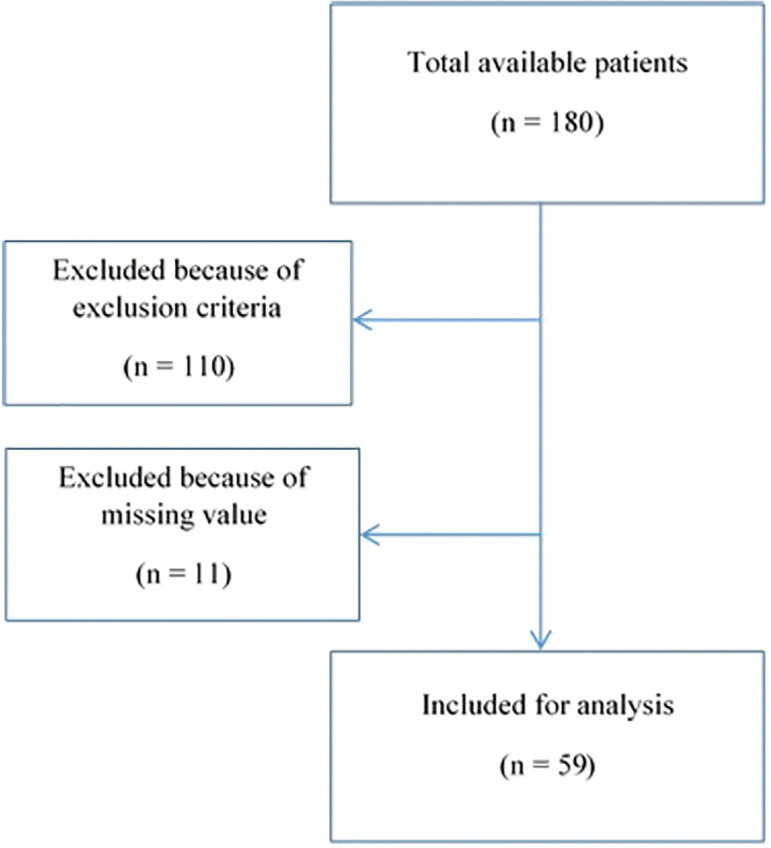
Flowchart of the study population

In the present prospective interventional study, we recruited 180 infertile men between 20-45 years of age, diagnosed with idiopathic OAT from Taleghani Hospital in Tehran, from 2017 to 2018. We included 70 patients in the study, according to the Gehan's formula. We obtained two semen samples from each patient: one sample before starting the antioxidant treatment and another sample after completing the antioxidant treatment. We took 11 patients off the study, because they did not return for the second analysis after the treatment (Figure 1). The Ethics Committee of the Shahid Beheshti University of Medical Sciences approved the study (IR.SBMU.RETECH.REC.1397.1359), and the trial received number IRCT20190426043379N1. All the participants signed a written informed consent before entering the study.

### Inclusion criteria

All the patients who received the treatment had more than one year of unprotected intercourse. The patients included in our study had at least one abnormal semen parameter (sperm concentration less than 15×10^6^ per ml, sperm motility less than 40%, and normal morphology less than 4%), according to the WHO criteria (World Health Organization, 2010). All the participants had less than 45 years of age, a BMI under 30, did not smoke, and had no addiction. An expert urologist visited all of them.

### Exclusion criteria

The exclusion criteria were azoospermia, prostatitis, genital trauma, testicular torsion, any genital disease (e.g., cryptorchidism, current genital inflammation, varicocele), endocrinopathy, any history of epididymo-orchitis, genital surgery, urinary tract infection, severe general or central nervous system disease, Y chromosome microdeletions or karyotype abnormalities, and recent sexually transmitted disease. In addition, we excluded the patients if they had any previous hormonal therapy or if they consumed cytotoxic drugs, alcohol drinking behaviors, drug abuse, immunosuppressants, anticonvulsive, androgens, antiandrogens, or any psychological or physiological abnormalities, which could impair their reproduction function or spermatogenesis. Also, having any diseases of the liver and biliary system, significant poor function of the kidneys, occupational and environmental exposures to potential reproductive toxins, and intolerance to the drug were other excluding factors.

### Study design

Before starting the treatment, a semen sample was collected from each patient after 3-5 days of abstinence. The samples were incubated for 20-30 min at 37ºC to be liquefied. After sample liquefaction, the semen parameters were determined according to WHO criteria (World Health Organization, 2010). For the antioxidant treatment, all patients received the Androferti supplement (Daru Darman Parmida, Iran) orally and twice daily. For detecting any effect of treatment on the semen parameters, the treatment continued for 3 months, because every spermatogenesis cycle period lasted for at least 3 months. The other semen sample was collected from each patient after the antioxidant supplementation treatment and analyzed together with the pre-treatment. Tolerability was evaluated based on the probable adverse effects reported by the patients and the physical examination conducted during the patient visit.

### Statistical analysis

We used the mean, median, standard deviation (SD), and interquartile range (IQR) to report the quantitative data. The qualitative data was represented using frequency and percentage. We used the non-parametric Wilcoxon signed-ranks test to compare data before and after treatment, with *p*<0.05 considered to be statistically significant. All the above-mentioned statistical analyses were carried out in SPSS software version 24.

## RESULTS

We had 59 infertile men, with at least one year of infertility accomplishing the treatment course. We analyzed the patients’ semen parameters and compared them based on pre-and post-antioxidant treatment. The patients had mean age of 34.5±4.16 (range: 26-40). Among all patients, 20.3% were in the age range of 26-30 years, 35.6% were between 31-35 years, and 44.1% were between 36-40 years of age. There was 100% treatment compliance among the patients. The mean marital age among the study subjects was 7.5±5.1 years (range: 1-21 years). Out of 59 patients, 11.9% had normal weight, 1.7% were underweight, and 84.6% were overweight. Table 1 depicts the baseline characteristics of the patients.

**Table 1:** Baseline characteristics of patients

Variable	Category	Frequency	Percent
**Age (years)**	26-30 31-35 36-40	12 21 26	20.3 35.6 44.1
**Job**	Self-employed Governmental	45 14	76.3 23.7
**Education level**	Illiterate Undergraduate Academic degree	2 39 14	3.4 82.8 13.8
**Body mass index (kg/m^2)^**	underweight Normal overweight	1 7 51	1.7 11.9 86.4
**Marriage age (years)**	≤ 5 6-10 11-15 ≥16	24 20 9 6	40.7 33.9 15.2 10.2

Table 2 shows that the concentration of sperm increased significantly after the antioxidants treatment (2.5 × 10^7^ [1.2 × 10^7^ - 5 × 10^7^] *vs.* 3.6 × 10^7^ [1.5 × 10^7^ - 7 × 10^7^], *p*=0.004) (Table 2). In addition, sperm morphology markedly increased after the treatment (1% [0% - 2%] *vs.* 1% [0% - 3%], *p*=0.013) (Table 2), although the increased value of sperm motility after treatment was not statistically significant. (28% [10% - 50%] *vs.* 35% [12% - 50%], *p*=0.2) (Table 2).

**Table 2:** Description and comparison of semen parameters before and after treatment

Semen parameter	Before treatment		After treatment		*p-*value
	Median	IQR	Median	IQR	
**Concentration (sperm /mL)**	2.5×10^7^	1.2×10^7^- 5×10^7^	3.6×10^7^	1.5×10^7^- 7×10^7^	0.004
**Morphology**	1%	0% - 2%	1%	0% - 3%	0.01
**Motility**	28%	10% - 50%	35%	12% - 50%	0.2

## DISCUSSION

Recent reports have indicated that semen quality has declined notably during the past 20 years ^(Alahmar, 2018)^, for which several causes have been discussed. Environmental factors (e.g., metal toxicity, chemicals, and other pollutants, radiation, heat, etc.), as well as obesity, inflammation, smoking, and ROS exposure, are counted as some causes of decreased spermatogenesis and sperm DNA integrity ^(Darbandi et al., 2018)^. Sperm quality and quantity - the determinative factors for male fertility - can be impacted by excessive ROS in the reproductive tract and semen. Therefore, sufficient antioxidant levels should be steadily maintained in the body to prevent excessive ROS from impairing spermatozoa proteins, lipid membranes, and DNA integrity. The present study demonstrates that antioxidants supplement containing 1500 mg L-carnitine can improve semen quality.

Previously, several supplements, including vitamins C and E, L-carnitine, coenzyme Q10, pentoxifylline, and trace elements (such as zinc and selenium), have been investigated for their conservative actions against ROS, individually or in combination. Accordingly, some of these antioxidant supplements have positively influenced sperm quality and quantity in the semen samples of participants, the DNA integrity of sperms, the total antioxidant capacity of seminal plasma, the success rate of pregnancy, and the *in vitro* fertilization outcomes ^(Agarwal et al., 2004^; ^Balercia et al., 2005^; ^Cavallini et al., 2004^; ^Lenzi et al., 2004^; ^Lombardo et al., 2011^; ^Rolf et al., 1999^; ^Comhaire et al., 2000^; ^Paradiso Galatioto et al., 2008^; ^Hargreave et al., 1984^; ^Vicari & Calogero, 2001^; ^Vicari *et al*., 2002^; ^Lenzi *et al*., 2003)^. In addition, there are reports of synergistically boosted positive effects from multi-therapy trials with antioxidants. However, the exact mechanism of the antioxidants’ role in conserving the sperm concentration has not yet been identified, the suppression of ROS-induced sperm damage has been repeatedly suggested as a probable mechanism ^(Agarwal *et al*., 2004^; ^Lombardo *et al*., 2011)^.

The present study showed that treatment with an edible antioxidant supplement (containing 1500 mg of L-Carnitine, 60 mg of vitamin C, 20 mg of coenzyme Q10, 10 mg of vitamin E, 10 mg of zinc, 200 µg of vitamin B9, 50 µg of selenium, 1 µg of vitamin B12) for 3 months could improve sperm concentration and morphology in infertile men with idiopathic AOT. Previously, in a study by ^Lenzi et al. (2004)^, they showed that a combined L-carnitine (2 g/d) and L-acetyl-carnitine (1 g/d) treatment for 2 months in infertile males with OAT increased sperm motility, but the other semen parameters did not improve in that study. In contrast to their findings, our study showed that antioxidant supplements containing L-carnitine (1500 mg) for 3 months could improve sperm concentration and morphology in men with idiopathic OAT, but not the sperm motility. In another study, oral antioxidant treatment containing 1500 mg of L-carnitine for 3 months increased sperm concentration, motility, and morphology in infertile patients with “known” OAT, which was not found in our study ^(Abad et al., 2013)^.

Nevertheless, there are also studies reporting no impact on semen parameters caused by antioxidants ^(Agarwal et al., 2004^; ^Lombardo et al., 2011)^. Comparing the compatible and opposite outcomes of various studies is challenging for several reasons. One reason is that many of such studies are randomized placebo-controlled trials or case-control studies, while others are performed under open, uncontrolled, or prospective methods. Antioxidants have also been studied in various doses and for varying durations, from 3 to 6 months, in various number of patients with idiopathic or known OAT. Another reason is that the negative results obtained from some similar studies can be interpreted as not enough reliable because of their small population size, insufficient doses of antioxidants, and/or short treatment duration ^(Agarwal *et al*., 2004)^. The root of antioxidant supplement administration also contributes to its effectiveness on the seminal fluid parameters, such that some studies report no appreciable improvement in sperm parameters by the oral administration of antioxidants ^(Agarwal *et al*., 2004^; ^Lombardo *et al*., 2011)^.

Regarding all the above-mentioned challenges and considering the reports that confirm the positive effects of antioxidant supplementation on sperm parameters, including the present one, we conclude that the dose and duration of treatment with antioxidants require being optimized in future related studies. We must also determine the seminal parameters that specifically benefit from a particular antioxidant and the efficacy of that specific antioxidative agent in mono- or poly-therapy. Also, adhering to a supplement treatment duration of at least 3 months, especially in cases of severe oxidative stress exposure, is an essential factor to be considered in studies since spermatozoa maturation takes around 72 days ^(Alahmar, 2018)^.

## CONCLUSIONS

According to the findings of the present study, a treatment intervention with antioxidant supplementation containing 1500 mg L-carnitine via oral administration can improve two sperm parameters in semen samples, including sperm concentration and morphology, while it did not change the sperm motility. Considering the demonstrated safety profile of L-carnitine supplementation, the present findings corroborate the oral supplementation with L-carnitine as a partially efficient intervention to improve the fertility in the idiopathic OAT male patients owing to its antioxidant potentials.
